# Two new species of the genus *Thornburghiella* Vaillant, 1982 (Diptera, Psychodidae, Psychodinae) from China

**DOI:** 10.3897/zookeys.1296.199004

**Published:** 2026-10-29

**Authors:** Shuailai Yang, Yining Xi, Pei Wang, Xinming Yin, Yuqiang Xi

**Affiliations:** 1 State Key Laboratory of Wheat and Maize Crop Science/Henan International Laboratory for Green Pest Control; Department of Entomology, Henan Agricultural University, No. 95 Wenhua Road, Jinshui District, Zhengzhou 450003, Henan Province, China State Key Laboratory of Wheat and Maize Crop Science/Henan International Laboratory for Green Pest Control; Department of Entomology, Henan Agricultural University Zhengzhou China; 2 Key Laboratory of Molluscan Quarantine and Identification of GACC, Fuzhou Customs District, No. 312 Hudong Road, Gulou District, Fuzhou 350001, Fujian Province, China Key Laboratory of Molluscan Quarantine and Identification of GACC Fuzhou China

**Keywords:** China, identification key, moth flies, new record, Pericomaini, Psychodidae, taxonomy, *
Thornburghiella
*

## Abstract

Two new species of the genus *Thornburghiella* Vaillant, 1982 collected from China, are described as new to science: *Thornburghiella sichuanensis***sp. nov**. and *Thornburghiella yintiaolingensis***sp. nov**. *Thornburghiella singularis* Quate, 1962 is recorded in China for the first time, and *Thornburghiella xinjiangensis* Wagner, 2003 is discovered in Inner Mongolia, China. An updated key to the species of *Thornburghiella* from China is presented.

## Introduction

Psychodinae comprise more than 2000 described species in more than 100 genera (Kvifte 2018). *Thornburghiella* Vaillant, 1982 belongs to Psychodinae, tribe Pericomaini (Kvifte 2018). The genus can be diagnosed based on the following characters: flagellomere 1 with a single row of 4–9 strong sensory spines, wings ovate without a sensory fold; apical flagellomere with apiculus shorter than base or absent; gonostyli mostly simple, conical, not completely bifurcated (sometimes forked with branches of varying shape); and aedeagal complex ovoid to angulate or divided into a pair of elongate, narrow, pointed or truncate extensions (Oboňa et al. 2017).

*Thornburghiella* was initially described by Vaillant (1973), but the name remained invalid because no type species was designated; the genus was validated in 1982, with *T. albitarsis* (Banks, 1895) designated as the type species (Vaillant 1982; Ježek 1992). Ježek (1992) began compiling a catalogue of *Thornburghiella*, which initially included 35 species. Oboňa et al. (2017) further expanded the number of known Palaearctic species of *Thornburghiella*, which, by the publication of Ježek et al. (2022), had increased to 47. Among them, Duckhouse (1987) treated *T. spinicornis* (Tokunaga, 1961) as a junior secondary homonym of *T. spinicornis* (Brunetti, 1908), for which he proposed the replacement name *T. tokunagai*. Both Duckhouse (1973) and Quate (1962) regarded *T. formosana* as a junior synonym of *T. spinicornis*; however, Duckhouse (1987) demonstrated that *T. spinicornis* (Brunetti, 1908) and *T. formosana* represent two distinct species based on morphological characters of the male genitalia. Following Curler and Moulton (2010), *T. spinicornis* is currently placed in the genus *Stupkaiella*.

Lastly, Kim and Kim (2025) added a new species from Korea. Therefore, as of now, the genus *Thornburghiella* comprises 49 species worldwide; over 40 of these are distributed in the Oriental and Palaearctic regions. Four species are known to occur in China (Tokunaga 1957; Wagner 2003): *T. globulipyga* Ježek, 1992, *T. kaszabi* (Vaillant, 1973), *T. xinjiangensis* Wagner, 2003 and *T. formosana* Tokunaga,1957.

In this study, two new species from China are described: *T. yintiaolingensis* sp. nov. and *T. sichuanensis* sp. nov.; *T. singularis* (Quate, 1962) is recorded in China for the first time; and *T. xinjiangensis* Wagner, 2003 is discovered in Inner Mongolia, China. A key to the known species of *Thornburghiella* from China is presented.

## Material and methods

Specimens were collected in the field using sweep netting; all collected specimens were immersed in absolute ethanol and preserved at −20 °C under frozen conditions. The head, wings, and male genitalia were dissected separately; genitalia preparations were made by removing and macerating the apical portion of the abdomen in glacial acetic acid, then rinsing in distilled water before storage in glycerin-filled microvials. After examination, the head, wings and genitalia were all preserved in small tubes filled with glycerol.

Specimens were examined and photographed using a Leica M205A stereomicroscope. Helicon Focus v. 7.0.2 was used for image stacking. Scale drawings were created based on the photographs, with partial structural details observed and refined under a stereomicroscope through adjustments and modifications before finalization. Photographs of the adult *in situ* were taken with a Sony a7m3 and Sony 90 mm macro lens, while environmental photos of the collection site were captured using a mobile phone camera (Redmi K60). Image plates were post-processed with Adobe Photoshop CC 2019 Extended.

All measurements of adults are in millimetres (mm). The palpus formula was calculated with the first segment as unit 1.

Specimens examined were deposited in the Entomological Museum of Henan Agricultural University (HAU), Zhengzhou. General morphological terminology follows that of Cumming and Wood (2017). Terminology for the wing follows Kvifte and Wagner (2017).

## Taxonomy

### Key to *Thornburghiella* species occurring in China

**Table:** 

1	Eye bridge separated by less than 2 facet diameters	***T. kaszabi* Vaillant, 1983**
–	Eye bridge separated by 3 or more facet diameters	**2**
2	Flagellomere 1 with row of 7 strong pines	**3**
–	Flagellomere 1 with row of 6 or 8 strong spines	**4**
3	Gonostyli shorter than gonocoxite, with no branch near its apex; surstylus apex and subapically with 46–48 tenacula	***T. globulipyga* Ježek, 1992**
–	Gonostyli longer than gonocoxite, with a single barbed branch subapically; surstylus apex bearing approximately 20–25 tenacula	***T. singularis* Quate, 1962**
4	Eye bridge of 5 facet rows	**5**
–	Eye bridge of more than 5 facet rows	**6**
5	Allurement organs S-shaped in lateral view, slightly enlarged at apex; gonocoxite swollen and nearly spherical; gonostyli smaller than gonocoxite; surstylus with tenacula clustered on proximal half and apex	***T. formosana* (Tokunaga, 1957)**
–	Allurement organs approximately oval-shaped in lateral view (Fig. 3E); gonostyli long, 1.8 times length of gonocoxite; tenacula occur only at apex of surstylus	***T. yintiaolingensis* sp. nov**.
6	Eye bridge of 7 facet rows; allurement organs oblong in lateral view (Fig. 5E); gonostyli cylindrical, apex sharply pointed, 1.72 times length of gonocoxite	***T. xinjiangensis* Wagner, 2003**
–	Eye bridge of 6 facet rows; allurement organs approximately boot-shaped in lateral view (Fig. 1E); gonostyli long, slender and flat (Fig. 1G), 2.56 times length of gonocoxites	***T. sichuanensis* sp. nov**.

#### Thornburghiella sichuanensis sp. nov.

Taxon classificationAnimaliaDipteraPsychodidae

0F341013-1BA2-514D-86E6-69214B24784D

https://zoobank.org/96675901-C5FF-471E-889B-7BF0D61BC4CA

Fig. 1A–H

##### Type material.

***Holotype***: • 1 ♂, China: Sichuan Province, Mianyang City, Wupin County, Wanglang National Nature Reserve, Changbaigou, 32°54'38.77"N, 104°9'25.13"E, 2403 m a.s.l., 2016.VII.17, leg. Yuqiang Xi. ***Paratypes***: • 1 ♂ China: same data as holotype.

##### Diagnosis.

Eye bridge of 6 facet rows; flagellomere 1 with row of 6 strong spines. Allurement organs boot-shaped. Aedeagus apex sharply pointed, rhombus-shaped; gonostyli long, slender and flat; surstylus apex with about 25 tenacula distally.

##### Description.

Measurements in mm (*N* = 1). **Male**. Body length 1.78 mm. Wing length 3.22 mm, width 1.35 mm. Head width 0.34 mm, length 0.35 mm; vertex 0.14 mm.

**Figure 1.**
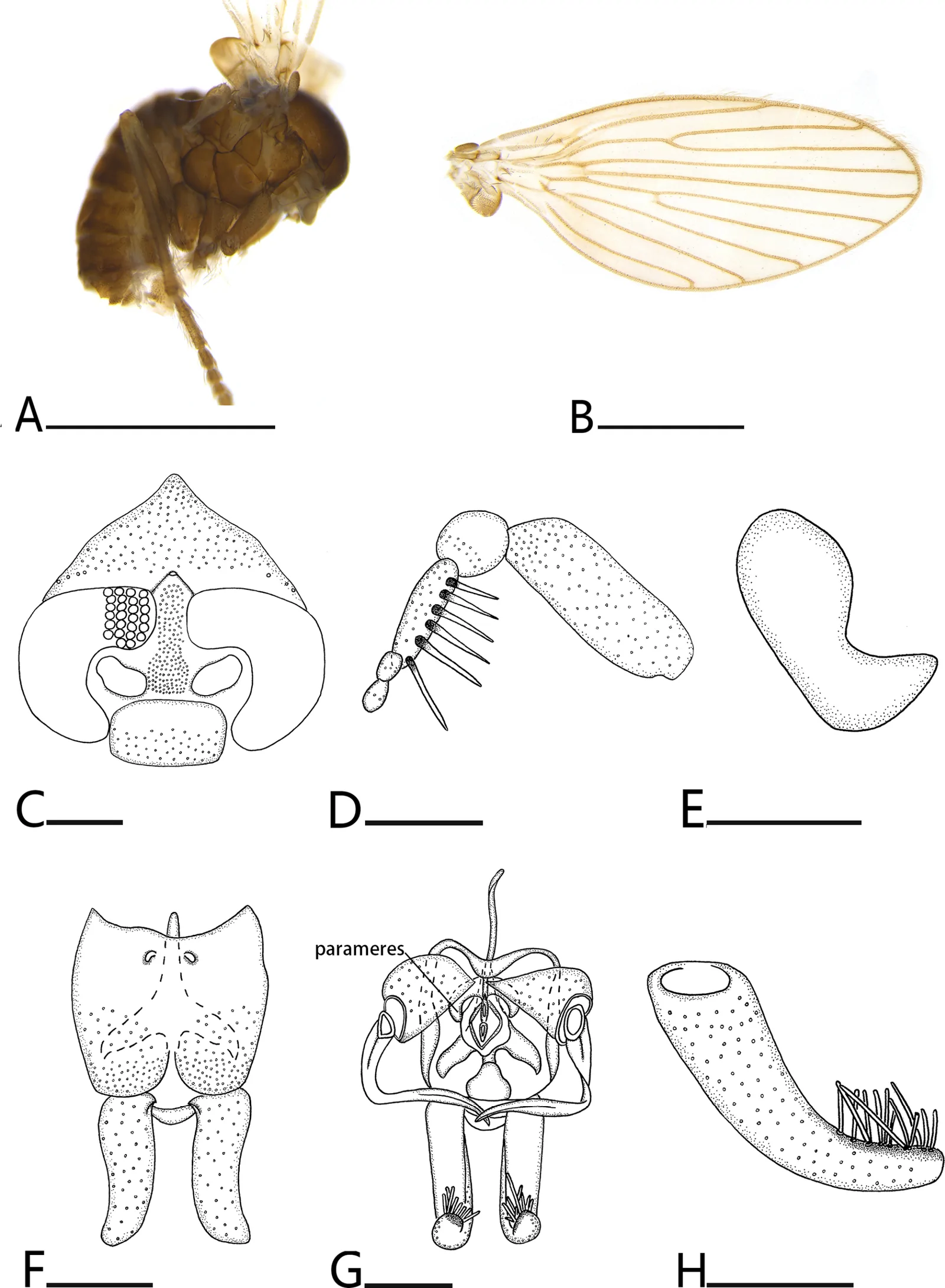
*Thornburghiella sichuanensis* sp. nov. **A**. Male, lateral view; **B**. Wing; **C**. Head; **D**. Antennae; **E**. Allurement organs, lateral view; **F**. Epandrium, dorsal view; **G**. Aedeagal complex; **H**. Surstylus, lateral view. Scale bars: 1 mm (**A, B**); 0.1 mm (**C–H**).

***Head***. (Fig. 1A) about the same length as width; vertex about 0.41 times length of head; with a row of ocular setae. Eye bridge of 6 facet rows, in the anterior three columns of the eye bridge, a few eye bridges exhibit seven facet rows, separated by about 5 facet diameters; interocular suture inverted, broadly V-shaped; frontal scar patch concentrated on inner margin of the antennal socket and extends to interocular suture. Antennae (Fig. 1D) 16 segments, scape length 0.18 mm, about 3.6 times the width, distally widened; pedicel rounded, length and width 0.05 mm; flagellomere 1 length 0.08 mm, with single row of 6 strong spines. Basal-most spine designated as the first spine, with successive lengths as follows: 1:0.04, 2:0.05, 3:0.05, 4:0.05, 5:0.06, 6:0.07. flagellomeres 4–13 broad fusiform, swollen at middle and narrowed distally; flagellomeres 14–15 rounded; flagellomere 16 rounded with an extension distally; ascoids short; length of antennae segments 1–16: 0.18: 0.05: 0.08: 0.02: 0.02: 0.02: 0.02: 0.02: 0.02: 0.02: 0.02: 0.02: 0.02: 0.02: 0.02: 0.03; Clypeus margin slightly flat, clypeus scar patch along outer margin clypeus. Palp segment proportions 1:1.64:1.86:2.29.

***Thorax*** with white allurement organs from the prothorax (Fig. 1E), approximately boot-shaped in lateral view, wide and round base, with constriction in the middle; anepimeron, anepisternum and katepisternum smooth; epimeron basal and medial half with setae. Wing (Fig. 1B) light brown; vein R_5_ situated below the apical angle of the wing; medial fork and radial fork are situated in the mid-wing region, with the medial fork positioned slightly more basally; medial fork slightly separated, and radial fork complete.

***Terminalia***. (Fig. 1D–F) Ejaculatory apodeme length 0.23 mm. Aedeagus apex sharply pointed, rhombus-shaped, enveloped by a transparent sac-like membrane; hypandrium slender, band-shaped, widened medially. Parameres (Fig. 1G) arc-shaped, surrounding the distiphallus. Gonocoxite length and width nearly equal, with many setae alveoli at apex margin; gonostyli long, slender and flat, length 0.23 mm, 2.56 times length of the gonocoxites, gonostyli sharply pointed at the apex and may sometimes be bent; epandrium rectangular, about same length as width, with scar patches covering nearly half of its surface, 2 pseudospiracular openings of epandrium small, irregular; hypoproct small in posterior view, semi-circular. Surstylus approximately 0.3 mm in lateral view, wider basally, narrowed toward apex; apex bearing about 25 tenacula distally, tenacula slender, varying in length, subtranslucent, the longest about 0.07 mm, the shortest about 0.03 mm.

**Female**. Unknown.

##### Distribution.

China (Sichuan).

##### Etymology.

The specific name “*sichuanensis*” refers to the type locality, Sichuan Province in China.

##### Remarks.

The new species resembles *T. melanderi* (Quate, 1955) in habitus, and both gonostyli and ejaculatory apodeme are long and slender, but it can be separated from the latter by the following: eye bridge separated by about 5 facet diameters; flagellomere 1 with a row of 6 strong spines; and the tenacula are distributed from the subapex to apex of the surstylus. In *T. melanderi*, the eye bridge is separated by about 2 facet diameters, flagellomere 1 has a row of 7 strong spines, and the tenacula are concentrated at the apex of the surstylus (Quate 1955).

#### Thornburghiella yintiaolingensis sp. nov.

Taxon classificationAnimaliaDipteraPsychodidae

9AAA654F-A61A-5BB5-9BEF-AA1256FDD85D

https://zoobank.org/E3FCF550-BC23-4595-A6B1-F1DD159CD35C

Figs 2A, 2B; 3A–H

##### Type material.

***Holotype***: • 1 ♂, China: Chongqing Municipality, Wuxi County, Yintiaoling National Nature Reserve, Guanshan Protection Station, Pianyanwu, 31°29'43.90"N, 109°42'45.63"E, 2410 m a.s.l., 2025.V.9, leg. Shuailai Yang. ***Paratypes***: • 1 ♂, China, same data as holotype.

##### Diagnosis.

Eye bridge of 5 facet rows, flagellomere 1 length bearing row of 6 long spines. Allurement organs approximately oval-shaped. Legs yellowish, femur at the base and tibia at apex dark brown; tarsus black. Gonostyli broader at the middle, slightly bent, forming an obtuse angle. Surstylus with 15–20 tenacula distally.

##### Description.

Measurements in mm (*N* = 1). **Male**. Body length 1.3 mm. Wing length 2.7 mm, width 1.1 mm. Head width 0.39 mm, length 0.38 mm; vertex 0.14 mm.

***Head***. (Fig. 3C) about the same length as width; vertex about 0.36 times length of head; without ocular setae. Eye bridge of 5 facet rows, separated by about 3.46 facet diameter; interocular suture inverted, broadly V-shaped; frontal scar patch concentrated on inner margin of the antennal socket and extending to interocular suture. Antennae (Fig. 3D) 16 segments, scape length 0.14 mm, about 2.05 times longer than wide; pedicel wide and rounded, subtriangular, about the same length as width; flagellomere 1 length 0.08 mm, with of 6 long spines, the basal spines 1–4 are equal in length, 0.061 mm, the fifth spine length 0.082 mm, the sixth spine length 0.11 mm, 1.81 times the length of spines 1–4; flagellomeres 2–13 fusiform, swollen at middle and narrowed distally; flagellomere 14 rounded with an extension at the distal; ascoids short; length of antennae segments 1–16: 0.14: 0.05: 0.08: 0.03: 0.03: 0.03: 0.03: 0.03: 0.03: 0.03: 0.03: 0.03: 0.02: 0.02: 0.02: 0.04; Clypeus margin slightly concave, clypeus scar patch along outer margin clypeus. Palp segment proportions 1:1.25:1.33:1.08

***Thorax*** dark brown with allurement organs from the prothorax (Fig. 3E) approximately oval-shaped in lateral view, anepimeron white and setae, anepisternum, katepisternum and epimeron smooth. Legs yellowish, femur at the base and tibia at apex dark brown; tarsus black. Wing (Fig. 3B) light brown; vein R_5_ situated below the apical angle of the wing; at the junction points where the wing veins (except for the Sc vein) meet the wing margin, the coloration is darker. Veins R_4,_ R_5_, and M_1_ intersect near the wing base, with a light gray spot present; medial fork and radial fork are situated in the mid-wing region, with the medial fork positioned slightly more basally, with the medial fork slightly separated, and the radial fork complete.

***Terminalia***. (Fig. 3F–H) Ejaculatory apodeme length 0.3 mm, narrow and long. Aedeagus apex triadius-shaped, enveloped by a transparent sac-like membrane; hypandrium slender, band-shaped. Parameres wide and flat, bending towards aedeagus; gonocoxite length and width nearly equal, with many scar patches at apex margin; gonostyli long and slender, pointed apex, slightly broader at the middle, slightly bent forming an obtuse angle, gonostyli length 0.18 mm, 1.8 times the length of the gonocoxite; epandrium rectangular, about the same length as width, with scar patches on nearly half of its surface, 2 pseudospiracular openings of epandrium small; hypoproct big and long, tongue-shaped in posterior view, broaden at the middle, with small setae at the apex; surstylus 0.17 mm in lateral view, base slightly widened, apex slender 15–20 tenacula distally, tenacula slender, varying in length, subtranslucent, the longest about 0.0988 mm, the shortest about 0.02 mm.

**Female**. Unknown.

##### Distribution.

China (Chongqing).

##### Etymology.

The specific name “*yintiaolingensis*” refers to the type locality, Yintiaoling National Nature Reserve, Chongqing Municipality in China.

##### Biology.

**Figure 2.**
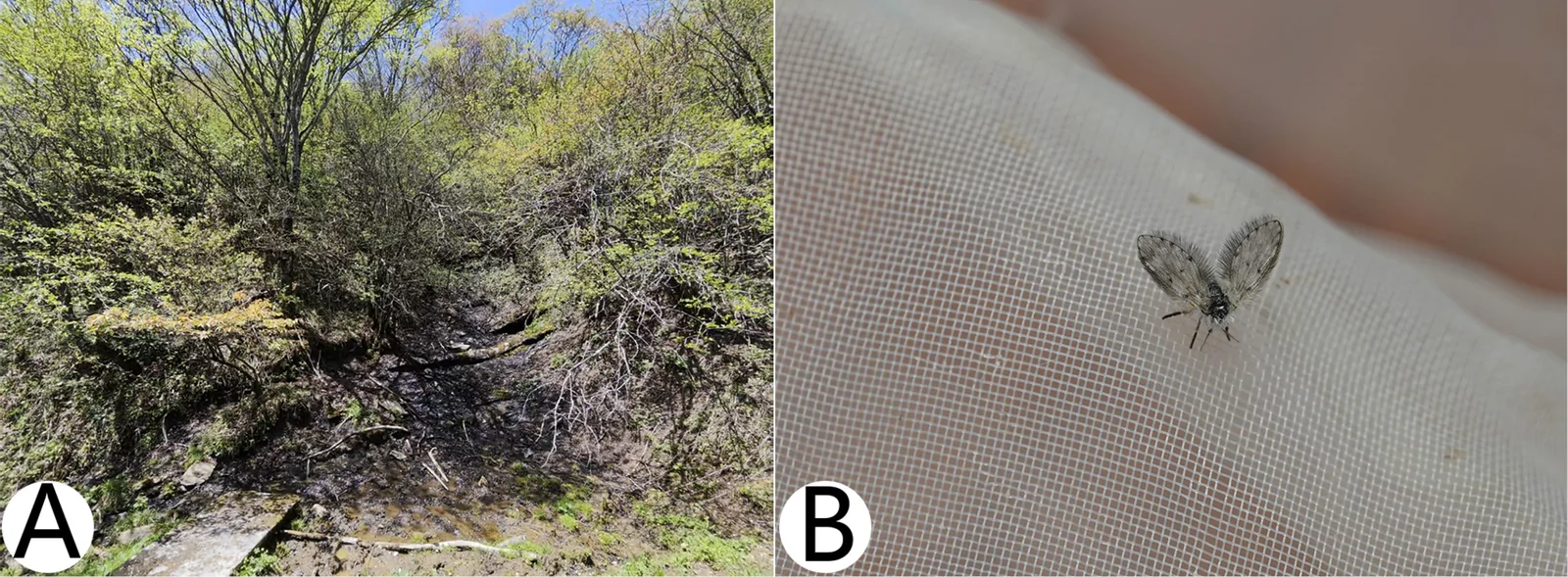
**A**. Type specimen collection site, Pianyanwu, Yintiaoling National Nature Reserve in Chongqing Municipality; **B**. Photograph of in situ *Thornburghiella yintiaolingensis* sp. nov.

The collection site was a small pool formed by converging streams, surrounded by abundant shrubs, and specimens were captured by sweep netting (Fig. 2A, B).

**Figure 3.**
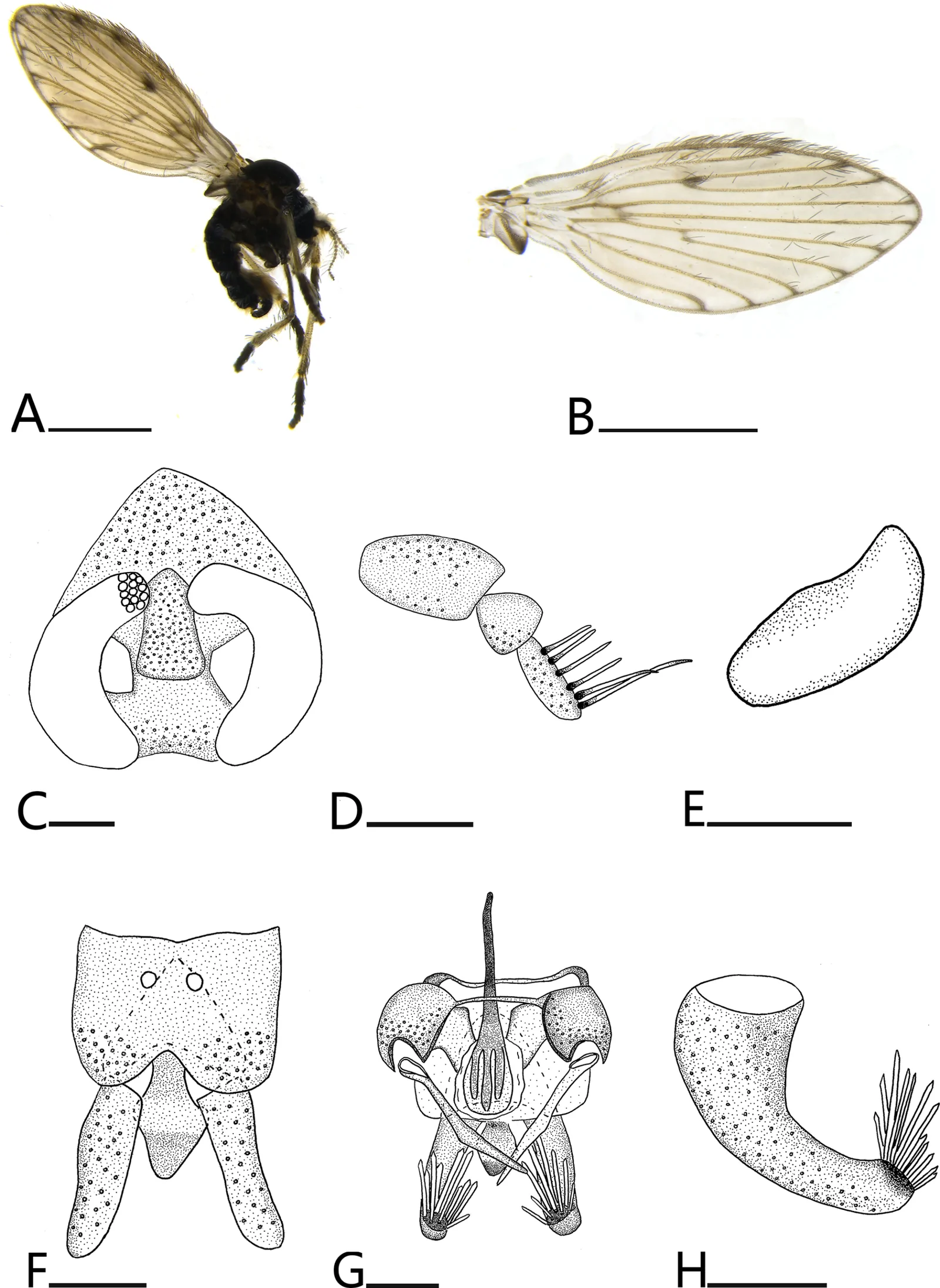
*Thornburghiella yintiaolingensis* sp. nov. **A**. Male, lateral view; **B**. Wing; **C**. Head; **D**. Antennae; **E**. Allurement organs, lateral view; **F**. Epandrium, dorsal view; **G**. Aedeagal complex; **H**. Surstylus, lateral view. Scale bars: 1 mm (**A, B**); 0.1 mm (**C–H**).

##### Remarks.

The new species resembles *T. itoi* (Tokunaga, 1961) in habitus; gonocoxite wide; gonostyli long and slender, pointed apex; tenacula concentrated at the apex of the surstylus, but it can be separated from the latter by the following: eye bridge separated by about 3.46 facet diameters; flagellomere 1 with a row of 6 strong spines; and gonostyli small at base. In *T. itoi* (Tokunaga, 1961), the eye bridge is separated by about 1.5 facet diameters; flagellomere 1 with a row of 5 strong spines; and gonostyli wide at base (Tokunaga 1961).

#### Thornburghiella singularis

Taxon classificationAnimaliaDipteraPsychodidae

(Quate, 1962)

D4A68BF5-F6FF-522C-89C3-FF69C9D4B828

Fig. 4A–H

Pericoma singularis Quate, 1962: 166
Thornburghiella singularis (Quate, 1962): Duckhouse 1987: 85

##### Material examined.

• 1 ♂, China, Tibet, Nyingchi City, Bayi, 29°38'11.67"N, 94°21'39.93"E, 2989 m a.s.l., 2017.VII.6, leg. Qicheng Yang.

##### Redescription.

Measurements in mm (*N* = 1). **Male**. Body length 2.25 mm. Wing length 3.6 mm, width 1.47 mm. Head width 0.61 mm, length 0.54 mm; vertex 0.21 mm.

**Figure 4.**
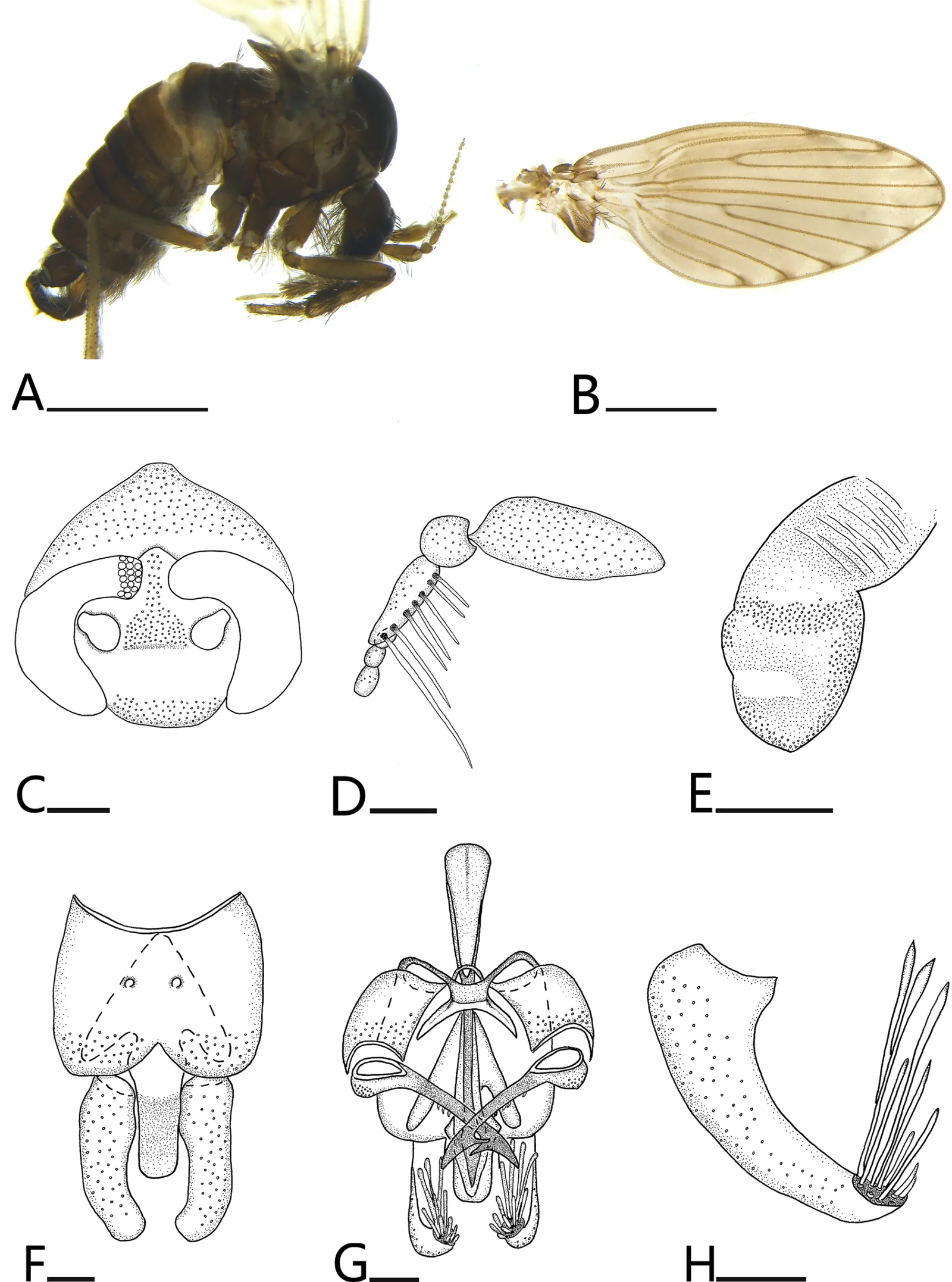
*Thornburghiella singularis* Quate, 1962. **A**. Adult, lateral view; **B**. Wing; **C**. Head; **D**. Antennae; **E**. Allurement organs, lateral view; **F**. Epandrium, dorsal view; **G**. Aedeagal complex; **H**. Surstylus, lateral view. Scale bars: 1 mm (**A, B**); 0.1 mm (**C–H**).

***Head***. (Fig. 4C) about the same length as width; vertex about 0.38 times length of head; without ocular setae. Eye bridge of seven facet rows, separated by about 4 facet diameters; interocular suture inverted, broadly V-shaped; frontal scar patch concentrated on inner margin of the antennal socket and extends to interocular suture. Antennae (Fig. 4D) 16 segments, scape length 0.3 mm, about 3 times longer than wide, distally widened; pedicel rounded, length 0.058 mm, width 0.066 mm; flagellomere 1 length 0.13 mm, bearing row of 7 strong spines. Basal-most spine is designated as the first spine, with successive lengths as follows: 1:0.07, 2:0.08, 3:0.09, 4:0.1, 5:0.11, 6:0.14, 7:0.22. The 7^th^ spine is longest, at 3.1 times length of 1^st^ spine; flagellomeres 2–13 fusiform, swollen at middle and narrowed distally; rounded flagellomere 14 with an extension at the distal half; ascoids short, length of antennae segments 1–16: 0.3: 0.06: 0.13: 0.04: 0.04: 0.04: 0.04: 0.04: 0.04: 0.04: 0.04: 0.04: 0.04: 0.03: 0.02: 0.06; Clypeus margin slightly flat, clypeus scar patch no more than half of clypeus. Palp segment proportions 1:1.7:1.8:2.6.

***Thorax*** with brown allurement organs from the prothorax (Fig. 4F), the distal end is slightly swollen in lateral view, with a setose surface and rugose at the base; epimeron, anepisternum and katepisternum smooth, anepimeron basal and medial half setae. Wing (Fig. 4B) light brown; vein R_5_ situated below the apical angle of the wing; dark brown spots present at the terminal junctions of all veins (except Sc) with the wing margin; medial fork and radial fork are situated in the mid-wing region, with the medial fork positioned slightly more basally, with the medial fork slightly separated and the radial fork complete.

***Terminalia***. (Fig. 4F–H). Ejaculatory apodeme length 0.29 mm, base narrow, long, broad at apex. Aedeagus apex sharply pointed, sword-shaped, surrounded by a translucent sac-like membrane; hypandrium slender, band-shaped, widened medially and bifurcate. Gonocoxite with many scar patches at apex margin, length and width nearly equal; gonostyli length 0.39 mm, 2.43 times the length of gonocoxite bearing multiple minute spines medially and a single barbed branch subapically, apex sharply pointed. Epandrium rectangular, width 1.25 times the length, with scar patches near the base, 2 pseudospiracular openings of epandrium small, irregular; hypoproct wide and long in posterior view, subrectangular; surstylus approximately 0.36 mm in lateral view, base slightly widened, apex slender; apex bearing approximately 20–25 tenacula distally, tenacula slender, varying in length, clustered, subtranslucent, the longest about 0.21 mm, the shortest 0.02 mm.

##### Distribution.

China (Tibet); India.

##### Remarks.

This specimen agrees with the original description in Quate (1962) of the species in all critical features: eye bridge with seven facet rows; first flagellomere bears a row of seven strong spines; thorax with large allurement organs; brown spots at the apices of wing veins; aedeagus long, spear-like; and gonocoxite bearing multiple minute spines medially. Only the shape of the hypandrium differs slightly. It is therefore confirmed as the same species and represents the first record of this species from China.

#### Thornburghiella xinjiangensis

Taxon classificationAnimaliaDipteraPsychodidae

Wagner, 2003

C0C40635-CEFC-52C8-8711-E2F31C9B48E7

Fig. 5 A–H

Thornburghiella xinjiangensis Wagner, 2003: 110

##### Material examined.

• 1 ♂, China, Inner Mongolia, Tongliao City, Ganqika Town, Horqin Left Rear Banner, Daqinggou National Nature Reserve, Headwaters of Daqinggou, 42°45'45"N, 122°12'14.33"E, 193 m a.s.l., 2014.VIII.20, leg. Yan Li. • 1 ♂, China, Inner Mongolia, Tongliao City, Ganqika Town, Horqin Left Rear Banner, Daqinggou National Nature Reserve, Headwaters of Daqinggou, 42°45'45"N, 122°12'14.33"E, 193 m a.s.l., 2014.VIII.27, leg. Li Shi.

##### Redescription.

Measurements in mm (*N* = 1). **Male**. Body length 2.1 mm. Wing length 3.1 mm, width 1.1 mm. Head width 0.52 mm, length 0.55 mm; vertex 0.16 mm.

**Figure 5.**
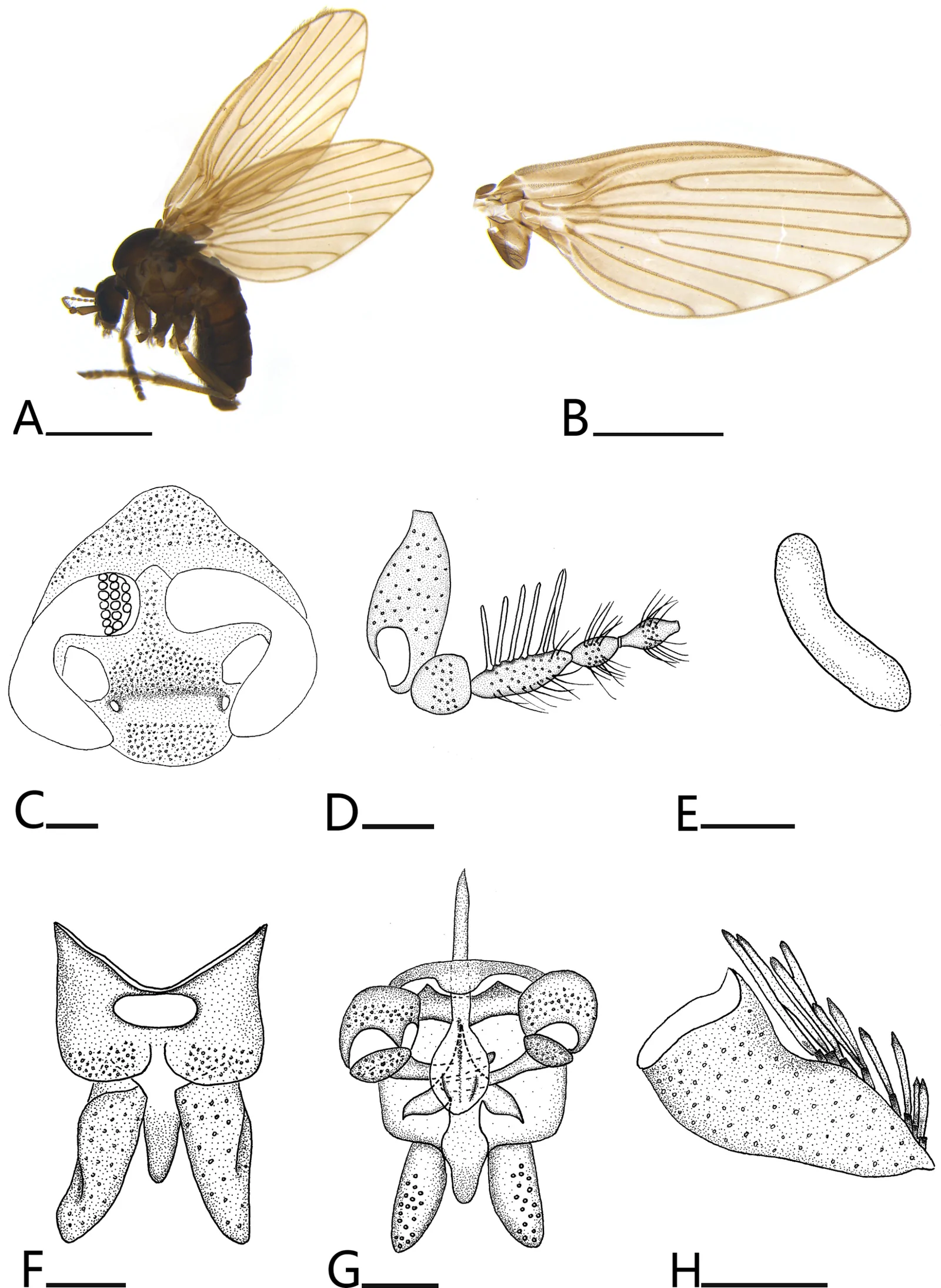
*Thornburghiella xinjiangensis* Wagner, 2003. **A**. Adult, lateral view; **B**. Wing; **C**. Head; **D**. Antennae; **E**. Allurement organs, lateral view; **F**. Epandrium, dorsal view; **G**. Aedeagal complex; **H**. Surstylus, lateral view. Scale bars: 1 mm (**A, B**); 0.1 mm (**C–H**).

***Head***. (Fig. 5C) about the same length as width; vertex about 0.308 times length of head; with a row of ocular setae. Eye bridge with 7 facet rows, separated by 3 facet diameters; interocular suture inverted, broadly V-shaped; frontal scar patch concentrated on the antennal socket and extended, but not reaching interocular suture. Antennae (Fig. 5D) 14 segments, scape length 0.2 mm, about 2.22 times longer than wide, distally widened; pedicel rounded, about the same length as width; flagellomere 1 length 0.12 mm, bearing row of 6 long spines. The first to fourth spines from the base are nearly equal in length, 0.07 mm, the fifth and sixth spines length 0.11 mm, which is 1.57 times the length of the first to fourth spines. Flagellomeres 4–13 fusiform, swollen at middle and narrowed distally; flagellomere 14 long cylindrical with an extension at the distal half; ascoids short; length of antennae segments 1–14: 0.2: 0.06: 0.12: 0.06: 0.06: 0.06: 0.06: 0.05: 0.05: 0.05: 0.04: 0.04: 0.04: 0.07; Clypeus margin slightly flat, clypeus scar patch no more than half of clypeus. Palp segment proportions 1:1.5:1.75:2.25 (44-60-62-70 in Wagner 2003).

***Thorax*** with brown allurement organs from the prothorax (Fig. 5E), short-stalked in lateral view; epimeron, anepisternum and katepisternum smooth, anepimeron basal and medial half setae. Wing (Fig. 5B) brown; vein R_5_ situated below the apical angle of the wing; dark brown spots present at the terminal junctions of all veins (except Sc) with the wing margin; The medial fork and radial fork are situated in the mid-wing region, with the medial fork positioned slightly more basally, with the medial fork slightly separated and the radial fork complete.

***Terminalia***. (Fig. 5F–H) Ejaculatory apodeme length 0.36 mm, narrow, long, broad at apex. Aedeagus with broad apex, surrounded by a translucent sac-like membrane; hypandrium slender, band-shaped, widened medially. Gonocoxite with many scar patches at apex margin, length and width nearly equal; gonostyli cylindrical, apex sharply pointed, base with dense clusters of scar patches. Gonostyli 0.19 mm in length, 1.72 times the length of the gonocoxite. Epandrium rectangular, width 1.47 times the length, with scar patches near the base, 1 big pseudospiracular opening of epandrium; hypoproct tongue-shaped in posterior view, broad at the base and gradually narrowing towards the apex. Surstylus approximately oblong, length 0.24 mm in lateral view, base slightly widened, apex slender; from the proximal half to the apex with approximately 16–19 tenacula (indicating that the number of tenacula is not perfectly symmetrical on both sides), varying in length, relatively dispersed, and inclined towards the base of the surstylus, slender and sharp at the apex, subtranslucent, the longest about 0.12 mm, the shortest 0.05 mm.

##### Distribution.

China (Inner Mongolia, Xinjiang).

##### Remarks.

The examined specimens agree with several important diagnostic characteristics of the original description in Wagner (2003) of this species: eye bridge 7 rows; antennae 14 segments; flagellomere 1 bearing a row of 6 long spines; thorax with short-stalked allurement organs; and surstylus from the proximal half to the apex with approximately 16–19 tenacula. Only the palpal ratio shows minor variation. This represents the first record of the species from Inner Mongolia, China.

## Discussion

Prior to this study, *Thornburghiella* was recorded only from Taiwan and Xinjiang provinces of China. With the new species and faunal records in the present work, the known distribution of this genus is further expanded to Sichuan, Inner Mongolia, Chongqing, and Xizang. Accordingly, *Thornburghiella* exhibits a wide distribution across China, spanning both northern and southern regions of the country. After the present study, *Thornburghiella* is known to comprise seven species in China: *T. formosana*, *T. globulipyga*, *T. kaszabi*, *T. xinjiangensis*, *T. singularis*, *T. sichuanensis* sp. nov., and *T. yintiaolingensis* sp. nov.

It is reasonable to infer that the actual species diversity of *Thornburghiella* in China remains underestimated, and further field investigations and taxonomic revisions are required to reveal additional undescribed species.

## Supplementary Material

XML Treatment for Thornburghiella sichuanensis

XML Treatment for Thornburghiella yintiaolingensis

XML Treatment for Thornburghiella singularis

XML Treatment for Thornburghiella xinjiangensis

## Additional information

### Artificial Intelligence (AI) use

The authors accept full responsibility for the content of the manuscript, including the disclosure of any use of AI.

No AI tools were used in the preparation of this manuscript.

### Author ORCIDs

S. Yang https://orcid.org/0009-0002-6813-8949

Y. Xi https://orcid.org/0009-0005-9515-0494

P. Wang https://orcid.org/0009-0007-8020-1492

X. Yin https://orcid.org/0000-0003-3936-3557

Y. Xi https://orcid.org/0000-0002-3285-8904

### Data availability

All of the data that support the findings of this study are available in the main text.
